# Dual C–Br Isotope Fractionation Indicates Distinct Reductive Dehalogenation Mechanisms of 1,2-Dibromoethane in *Dehalococcoides*- and *Dehalogenimonas*-Containing Cultures

**DOI:** 10.1021/acs.est.2c07137

**Published:** 2023-01-26

**Authors:** Jordi Palau, Alba Trueba-Santiso, Rong Yu, Siti Hatijah Mortan, Orfan Shouakar-Stash, David L. Freedman, Kenneth Wasmund, Daniel Hunkeler, Ernest Marco-Urrea, Monica Rosell

**Affiliations:** † Grup MAiMA, SGR Mineralogia Aplicada, Geoquímica i Geomicrobiologia, Departament de Mineralogia, Petrologia i Geologia Aplicada, Facultat de Ciències de la Terra, Institut de Recerca de l’Aigua (IdRA), Universitat de Barcelona (UB), Martí Franquès s/n, Barcelona08028, Spain; ‡ Departament d’Enginyeria Química, Biològica i Ambiental, Universitat Autònoma de Barcelona (UAB), Carrer de les Sitges s/n, Bellaterra08193, Spain; § Synterra Corporation, Greenville, South Carolina29601, United States; ∥ Isotope Tracer Technologies Inc., Waterloo, OntarioN2V 1K4, Canada; ⊥ Department of Environmental Engineering and Earth Sciences, Clemson University, Clemson, South Carolina29634, United States; # Division of Microbial Ecology, Centre for Microbiology and Environmental Systems Science, University of Vienna, ViennaA-1030, Austria; ¶ Centre for Hydrogeology and Geothermics, University of Neuchâtel, Neuchâtel2000, Switzerland

**Keywords:** brominated organic compounds, groundwater contamination, biodegradation, organohalide-respiring bacteria, compound-specific isotope analysis

## Abstract

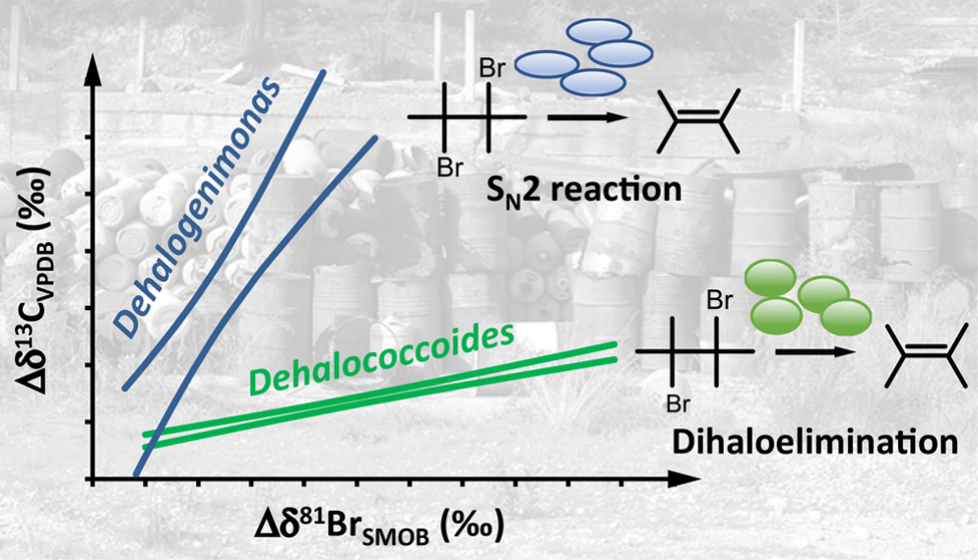

Brominated organic compounds such as 1,2-dibromoethane (1,2-DBA) are highly toxic groundwater contaminants. Multi-element compound-specific isotope analysis bears the potential to elucidate the biodegradation pathways of 1,2-DBA in the environment, which is crucial information to assess its fate in contaminated sites. This study investigates for the first time dual C–Br isotope fractionation during in vivo biodegradation of 1,2-DBA by two anaerobic enrichment cultures containing organohalide-respiring bacteria (i.e., either *Dehalococcoides* or *Dehalogenimonas*). Different ε_bulk_^C^ values (−1.8 ± 0.2 and −19.2 ± 3.5‰, respectively) were obtained, whereas their respective ε_bulk_^Br^ values were lower and similar to each other (−1.22 ± 0.08 and −1.2 ± 0.5‰), leading to distinctly different trends (Λ_C–Br_ = Δδ^13^C/Δδ^81^Br ≈ ε_bulk_^C^/ε_bulk_^Br^) in a dual C–Br isotope plot (1.4 ± 0.2 and 12 ± 4, respectively). These results suggest the occurrence of different underlying reaction mechanisms during enzymatic 1,2-DBA transformation, that is, concerted dihaloelimination and nucleophilic substitution (S_N_2-reaction). The strongly pathway-dependent Λ_C–Br_ values illustrate the potential of this approach to elucidate the reaction mechanism of 1,2-DBA in the field and to select appropriate ε_bulk_^C^ values for quantification of biodegradation. The results of this study provide valuable information for future biodegradation studies of 1,2-DBA in contaminated sites.

## Abstract 2

New insights into the reaction mechanisms of microbial reductive dehalogenation of brominated ethanes and information relevant to the application of CSIA in field studies.

## Introduction

1,2-Dibromoethane (1,2-DBA), also known as ethylene dibromide, is highly toxic (U.S. EPA drinking water maximum contaminant level, MCL, 0.05 μg/L)^1^ and persistent in the environment.^2^ It is a suspected human carcinogen that was used as a lead scavenger in gasoline, as well as a pesticide, fumigant, solvent, and chemical intermediate.^2^ As a result of its extensive use, 1,2-DBA has been detected above its MCL in groundwater samples from domestic wells in the USA^3^ and in about half of the contaminated underground storage tank sites in South Carolina (1100 sites evaluated).^4,5^ It is ranked 39th (out of 275) on the 2019 substance priority list established by the U.S. Agency for Toxic Substances and Disease Registry based on a combination of its frequency, toxicity, and potential for human exposure.^6^ Therefore, subsurface contamination by 1,2-DBA is an issue of environmental concern.

**Scheme 1:**
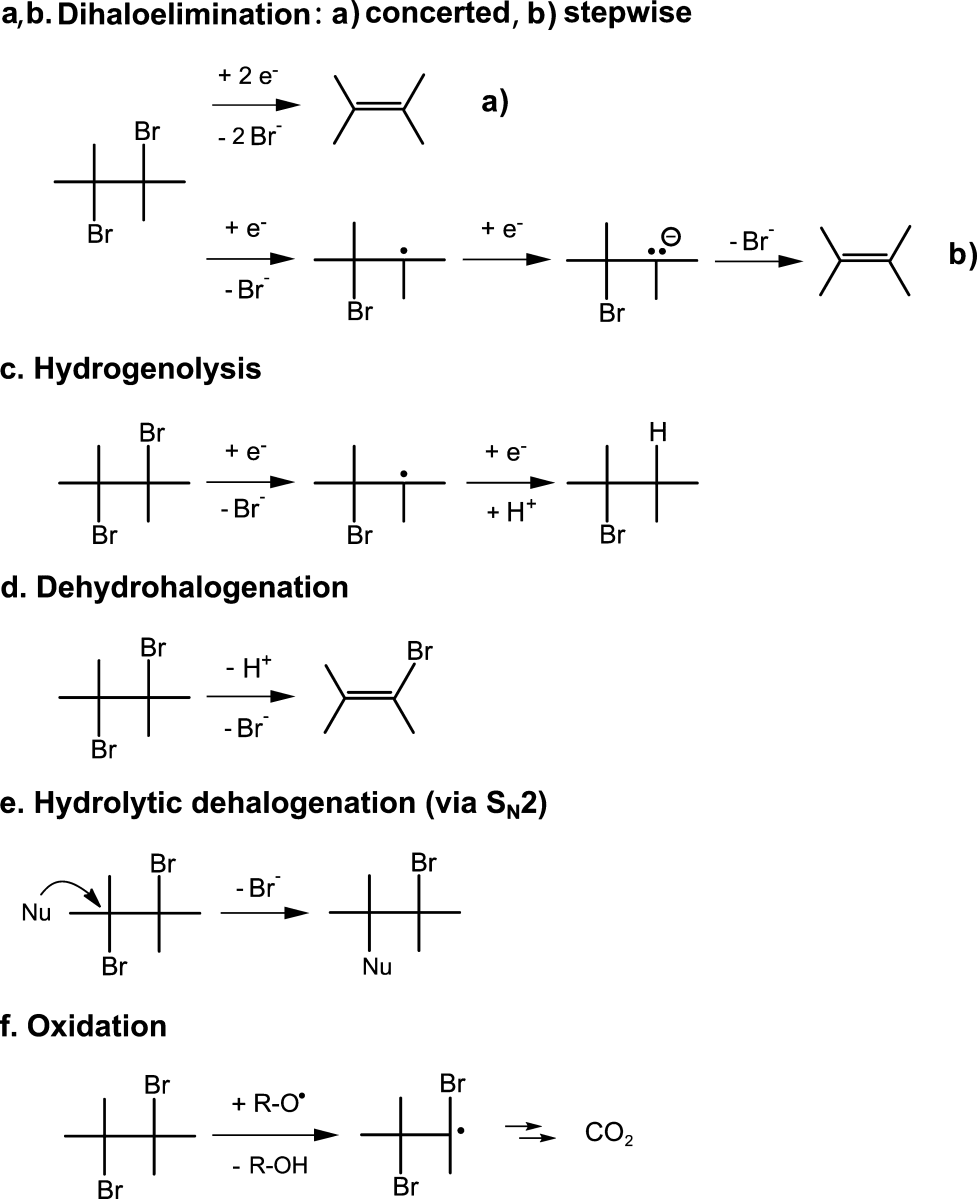
Biodegradation Pathways of 1,2-DBA Dots in structural formulas represent unpaired electrons.

At contaminated sites, anoxic conditions are prevalent in groundwater due to depletion of oxygen during degradation of readily oxidizable organic contaminants such as petroleum hydrocarbons, which are often detected at field sites impacted by 1,2-DBA due to its use as a lead scavenger. Organohalide-respiring bacteria (OHRB) provide a potential solution to treat 1,2-DBA-impacted sites due to their capability to harness energy using halogenated compounds as electron acceptors.^7^ During this organohalide respiration process, 1,2-DBA is predominantly transformed to innocuous ethene.^5^ Formation of ethene can occur via dihaloelimination of 1,2-DBA, either via *concerted* or *stepwise* β-elimination (Scheme 1a,b). Hydrogenolysis to bromoethane (Scheme 1c)^8^ and formation of small amounts of vinyl bromide (VB) via dehydrohalogenation (Scheme 1d)^5,9^ were reported in previous anaerobic biodegradation studies.

In addition to reductive dehalogenation and dehydrohalogenation reactions, 1,2-DBA can be transformed via nucleophilic substitution (S_N_2) to 2-bromoethanol (Scheme 1e) as an intermediate product under oxic conditions.^10,11^ Oxidation of 1,2-DBA catalyzed by a monooxygenase enzyme was also suggested in previous studies (Scheme 1f).^12,13^ Thus, the high susceptibility of 1,2-DBA to being transformed via distinct biodegradation pathways, under both anoxic and oxic conditions, complicates the assessment of its fate in the environment. Understanding the route of biodegradation is important to evaluate the natural attenuation of 1,2-DBA at contaminated sites and to predict the potential for accumulation of toxic halogenated daughter products in the field.

Identifying degradation pathways from analysis of daughter products in field samples is complex since it requires that initial contaminants and degradation products are adequately captured by the sampling and analysis methods. Furthermore, products can be either (i) rapidly converted (e.g., ethene or 2-bromoethanol^10^) to ubiquitous end products or (ii) formed (e.g., VB or ethene) from different precursors. For instance, ethene can be formed from brominated and chlorinated ethenes via hydrogenolysis. Hence, additional tools are necessary for better characterization of 1,2-DBA biodegradation in the field.

Compound-specific isotope analysis (CSIA) is a powerful tool to assess transformation processes of organic contaminants.^14,15^ The ratio of the heavy to light isotopes of an element in a sample *R*_sample_ (e.g., ^13^C/^12^C or ^81^Br/^79^Br) is expressed using the δ-notation as per mil (‰) difference from the isotope ratio in the respective standard *R*_standard_, that is
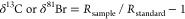
1

Changes in isotope ratios during a specific transformation of a compound can be described by a modified form of the Rayleigh distillation equation
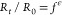
2

The compound-specific isotope fractionation value (ε) is related to the initial isotope ratio at the beginning of a transformation process *R*_0_, the isotope ratio *R*_*t*_, and the compound remaining fraction (*f* = *C*_*t*_/*C*_0_) at a given time point.

For carbon, a wide range of ε_bulk_^C^ (molecular-average isotope effects) values were observed during transformation of 1,2-DBA to ethene in previous laboratory studies, from −5.3 ± 0.5 to −20 ± 3‰.^11,13^ Such variability can reflect not only the occurrence of rate-limiting (non- or slightly isotope-fractionating) steps preceding the bond cleavage, such as contaminant mass transfer^16^ (so called isotope masking), but also the effect of different reaction mechanisms (e.g., stepwise vs concerted dihaloelimination, Scheme 1a,b), which hampers unambiguous identification of the reaction mechanism.

The situation is different if isotope analysis is conducted on two or more elements (e.g., C and Br). Combined changes in isotope ratios of both elements (e.g., Δδ^13^C vs Δδ^81^Br) generally exhibit a linear relationship with slopes (Λ_C–Br_ = Δδ^13^C/Δδ^81^Br ≈ ε_bulk_^C^/ε_bulk_^Br^), reflecting the extent of C and Br isotope effects,^11^ which relate to the respective underlying reaction mechanisms.^15,17^ In contrast to single-element isotope fractionation analysis, the proportion of changes in isotope ratios of both elements relative to each other (Δδ^13^C/Δδ^81^Br) is largely insensitive toward non-degradative processes such as contaminant transport.^18−20^ However, Br isotope fractionation data and dual C–Br isotope fractionation studies are very limited so far.^21^

Using a multi-element CSIA approach, dual-isotope fractionation patterns observed at contaminated sites can be compared to the laboratory-derived Λ values in order to identify degradation pathways in the field.^22,23^ A recent study by Kuntze et al.^11^ reported Λ_C–Br_ values determined during debromination of 1,2-DBA to ethene by reduced corrinoids (norpseudo-B_12_ and cyano-B_12_ types) and crude protein extracts of OHRB *Sulfurospirillum multivorans*. In contrast to this previous study, Λ_C–Br_ values have not yet been reported for in vivo biodegradation of 1,2-DBA by OHRB such as *Dehalococcoides* spp. or *Dehalogenimonas* spp., which is necessary to evaluate whether the reaction mechanisms and isotope fractionation effects determined in vitro are also at work in living organisms. In addition, previous laboratory isotope studies on biodegradation of chlorinated ethenes showed a significant difference of ε_bulk_^C^ in experiments with corrinoids versus microbial cultures^24^ and also between cultures and crude extracts.^16^ Thus, determining C and Br isotope fractionation and dual-element isotope slopes in in vivo experiments is warranted. This information is not only important for process understanding but also essential when using Λ and ε_bulk_ values to assess the fate and to quantify the biodegradation extent of 1,2-DBA in the environment.

Biodegradation of 1,2-DBA by different reductive dehalogenases (RDases) was reported in previous studies. The trichloroethene RDase (TceA) from *Dehalococcoides mccartyi* strain 195 converted 1,2-DBA to ethene and minor amounts of VB (<1%) in enzymatic assays with the purified enzyme.^9^ More recently, debromination of 1,2-DBA to ethene was catalyzed by tetrachloroethene RDase (PceA) in enzymatic assays with crude protein extracts from *S. multivorans*.^11^ However, in these studies, it was not investigated if 1,2-DBA would support the growth of the organisms. For *Dehalococcoides*, Yu et al.^5^ showed that the number of *D. mccartyi* cells in an anaerobic enrichment culture increased with 1,2-DBA amendments, producing predominantly ethene and traces of VB. Bacteria of the genus *Dehalogenimonas* are known for their potential to grow by reductive dehalogenation with vicinal-halogenated alkanes.^25^ Despite this, very little information is available on the biodegradation of 1,2-DBA by *Dehalogenimonas*.^26^

The main aim of the present study was to investigate for the first time (i) dual C–Br isotope fractionation during in vivo biodegradation of 1,2-DBA and (ii) whether distinct or similar dual-element isotope slopes occur with different bacteria. To this end, carbon and bromine isotopic fractionation (ε_bulk_^C^ and ε_bulk_^Br^) for microbial reductive dehalogenation of 1,2-DBA were determined using two laboratory cultures enriched in *Dehalococcoides* and *Dehalogenimonas* populations, respectively. A dual C–Br isotope approach was used to characterize the Λ_C–Br_ value and explore the underlying reaction mechanism. The determined Λ_C–Br_ values from in vivo experiments with intact cells of *Dehalococcoides* and *Dehalogenimonas*, respectively, were compared with those reported for 1,2-DBA degradation by corrinoids and crude protein extracts (*S. multivorans*).^11^ In addition, the biodegradation of 1,2-DBA by the *Dehalogenimonas*-containing culture used in this study was characterized.

## Materials and Methods

### Bacterial Cultures

Two enrichment cultures with different bacterial populations were used for in vivo laboratory batch experiments with respiring cells. The *Dehalogenimonas*-containing culture used in this study was derived from sediments obtained from the Besòs River (Spain); the genome of this strain was recently sequenced and annotated, and it was denominated *Dehalogenimonas alkenigignens* strain BRE15M.^27^ The composition of this consortium is shown in the Supporting Information (Figure S1). Further information regarding enzymatic assays with cell suspensions, cell harvesting, DNA extraction, and 16S rRNA gene amplicon sequencing is provided in Supporting Information.

The *Dehalococcoides*-containing enrichment culture was derived from samples collected from the Savannah River site (USA), and previous studies have focused on its kinetics and yields.^5^ Growth of *Dehalococcoides* occurred when the culture used 1,2-DBA as the electron acceptor and lactate as the electron donor.^5^ For both cultures, details regarding bacterial cultivation are provided in the Supporting Information.

### Isotope Fractionation Experiments with *Dehalococcoides* and *Dehalogenimonas* Cultures

The chemicals and medium used for the preparation of microcosms, incubation conditions, and sampling details are described in the Supporting Information. Batch tests with *Dehalococcoides-* and *Dehalogenimonas*-containing microcosms were performed at Clemson University (CU), USA, and at the Universitat Autònoma de Barcelona (UAB), Spain, respectively. Serum bottles (120 mL total volume) containing 65 mL of medium were prepared in an anoxic chamber and sealed with Teflon-faced rubber septa and aluminum crimp caps to maintain anoxic conditions. Initial aqueous phase concentrations of 1,2-DBA (considering partitioning between the headspace and the liquid using Henry’s law) were ∼270 and ∼25 μM for experiments with *Dehalococcoides* and *Dehalogenimonas*, respectively. Bottles were sacrificed at different extents of debromination by adding concentrated phosphoric acid (300 μL), as described elsewhere.^23,28^ For the experiments performed at CU, killed controls were prepared by adding phosphoric acid first to the bottles, followed by 1,2-DBA. For those performed at UAB, abiotic control bottles containing the growth medium with 1,2-DBA but without inoculum were prepared, as described for the experimental bottles. In addition, live controls without 1,2-DBA were prepared to account for the transfer of compounds from previous degradation experiments with the inoculum.

### Concentration and Isotopic Analyses

A detailed description of analytical methods and equipment used for the isotopic and concentration analysis is available in the Supporting Information. The concentrations of 1,2-DBA and daughter products (i.e., ethene and VB) were measured by headspace analysis using a gas chromatograph-flame ionization detector at CU^5^ and UAB^29^ laboratories (see the Supporting Information). The concentration of 1,2-DBA in abiotic controls of the experiments with *Dehalococcoides* (270 ± 3 μM, *n* = 3) and *Dehalogenimonas* (26 ± 2 μM, *n* = 2) containing microcosms remained at the initial concentration, indicating that compound losses through the caps during incubation were insignificant.

Bromine isotope measurements of 1,2-DBA were performed at Isotope Tracer Technologies Inc. (IT2), Canada, and carbon isotope ratios were analyzed at the Scientific and Technological Centers of the University of Barcelona (CCiT-UB), Spain. ^81^Br- and ^13^C-CSIA were performed by gas chromatography–isotope ratio mass spectrometry (GC–IRMS). For bromine, the two most abundant fragment ions (*m*/*z* 109 and 107) were used, which correspond to isotopologue pairs that differ by one heavy bromine isotope ([^81^Br^12^C_2_^1^H_4_]^+^ and [^79^Br^12^C_2_^1^H_4_]^+^, respectively). The raw δ^81^Br values were calibrated to the standard mean ocean bromide scale using two external laboratory standards of 1,2-DBA (two-point calibration), which were dissolved in water and measured similarly to the samples in the same sequence. Duplicate samples and standards were analyzed as a quality control for both C and Br isotopes, and the precision (±1σ) of the analysis was ≤ ±0.3‰ for both δ^13^C and δ^81^Br.

### Evaluation of Isotope Fractionation

The compound-average ε_bulk_ values were quantified by least-squares linear regression of eq 2 without forcing the regression through the origin,^30^ and the uncertainty corresponds to the 95% confidence interval (C.I.) derived from the standard deviation of the regression slope. Like ^37^Cl (natural abundance of 24.22%), the heavy bromine stable isotope (^81^Br) also has a high abundance (40.3%).^31^ A previous study^32^ showed that the Rayleigh equation can also be applied to calculate the isotopic fractionation of chlorine despite the higher natural abundance of ^37^Cl compared to ^13^C, and this equation was recently used in Br isotope fractionation studies with different compounds (e.g., 1,2-DBA,^11^ methyl bromide,^33^ and brominated ethenes^34^). Calculation of C and Br apparent kinetic isotope effects (AKIEs) from estimated ε_bulk_ values is presented in the Supporting Information, and their uncertainty was calculated by error propagation.

## Results and Discussion

### Reductive Dehalogenation of 1,2-DBA by Enrichment Cultures Containing *Dehalogenimonas* and *Dehalococcoides* Species

Metagenomic analyses determined that the bacterial community in the *Dehalogenimonas*-containing culture was dominated by the genus *Dehalogenimonas* (68.0%, Figure S1) and did not detect any other OHRB belonging to the class *Dehalococcoidia*. Importantly, the absence of *Dehalococcoides* spp. was previously corroborated in this culture by specific PCR assays for the genus *Dehalococcoides*.^29^

**Figure 1:**
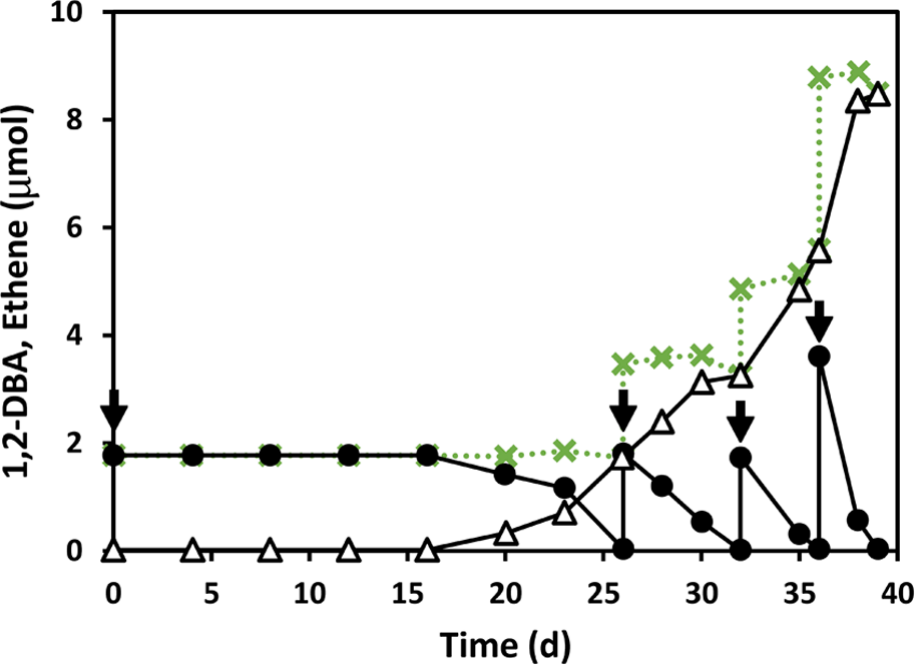
Debromination of 1,2-DBA to ethene in the first generation of *Dehalogenimonas*-containing culture cultivated with this halogenated substrate. Arrows indicate additional amendments of 1,2-DBA. Black circles correspond to micromoles of 1,2-DBA left in the microcosms, white triangles to micromoles of ethene, and green crosses to the total amount of 1,2-DBA and ethene. Error bars representing the standard deviation for the values of biological triplicates are smaller than the symbols.

Debromination of 1,2-DBA to ethene was observed after transferring the *Dehalogenimonas*-containing culture to a medium amended with 1,2-DBA (25 μM) as the sole halogenated electron acceptor (Figure 1). After a lag phase, the initial concentration of 1,2-DBA was depleted, and this was followed by consumption of additional amendments with increasing degradation rates, suggesting microbial growth coupled to the biodegradation of 1,2-DBA. After consumption of 100 μM 1,2-DBA, quantitative real-time PCR from technical triplicates of two experimental microcosms showed that *Dehalogenimonas* 16S rRNA gene copies doubled from 10.4 × 10^4^ ± 2.9 × 10^4^ (at time zero) to 22.3 × 10^4^ ± 1.5 × 10^4^, suggesting again that reduction of 1,2-DBA was coupled to growth. To the best of our knowledge, this is the first evidence that the *Dehalogenimonas* genus can couple its growth with 1,2-DBA dehalogenation.

Degradation products of 1,2-DBA (i.e., ethene and VB) were analyzed with *Dehalogenimonas* and *Dehalococcoides* cultures, as this can provide important information on the enzymatic reaction mechanism. In the experiments with the *Dehalogenimonas*-containing culture, only ethene and bromide were detected as a result of 1,2-DBA transformation, and they were produced at stoichiometric amounts (Figure S2). VB was never detected (detection limit, 0.5 μM). Reductive dehalogenation of 1,2-DBA (initial concentration of 50 μM) to ethene was corroborated using enzymatic assays with cell suspensions and methyl viologen as the artificial electron donor (Table S1). In addition, VB was not transformed in enzymatic assays with cell suspensions using the *Dehalogenimonas*-containing culture grown with 1,2-DBA, ruling out a hypothetical formation of ethene via reduction of VB as the intermediate.

For the experiments with the *Dehalococcoides*-containing culture, time-course concentration data during 1,2-DBA biodegradation are shown in Figure S3. Small amounts of VB (less than 0.2%, in mol/bottle basis, of the initial 1,2-DBA added) were detected in experiments with the *Dehalococcoides*-containing culture (Figure S3), in agreement with previous biodegradation studies of 1,2-DBA by *Dehalococcoides* showing transformation of 1,2-DBA to ethene and minor amounts of VB (<1%).^5,9^ The amount of VB measured in this study was much higher than that expected for abiotic dehydrohalogenation of 1,2-DBA (see Results in Supporting Information and Figure S3) and, therefore, this abiotic pathway is not a plausible explanation for the origin of VB. The product concentration pattern in Figure S3 indicates 1,2-DBA transformation to ethene as the main degradation pathway, as observed for *Dehalogenimonas*. The low level of VB that accumulated could be subsequently reduced in part to ethene according to the decreasing amounts of VB toward the end of the experiment (Figure S3).

### Br and C Isotope Fractionation Experiments

**Figure 2:**
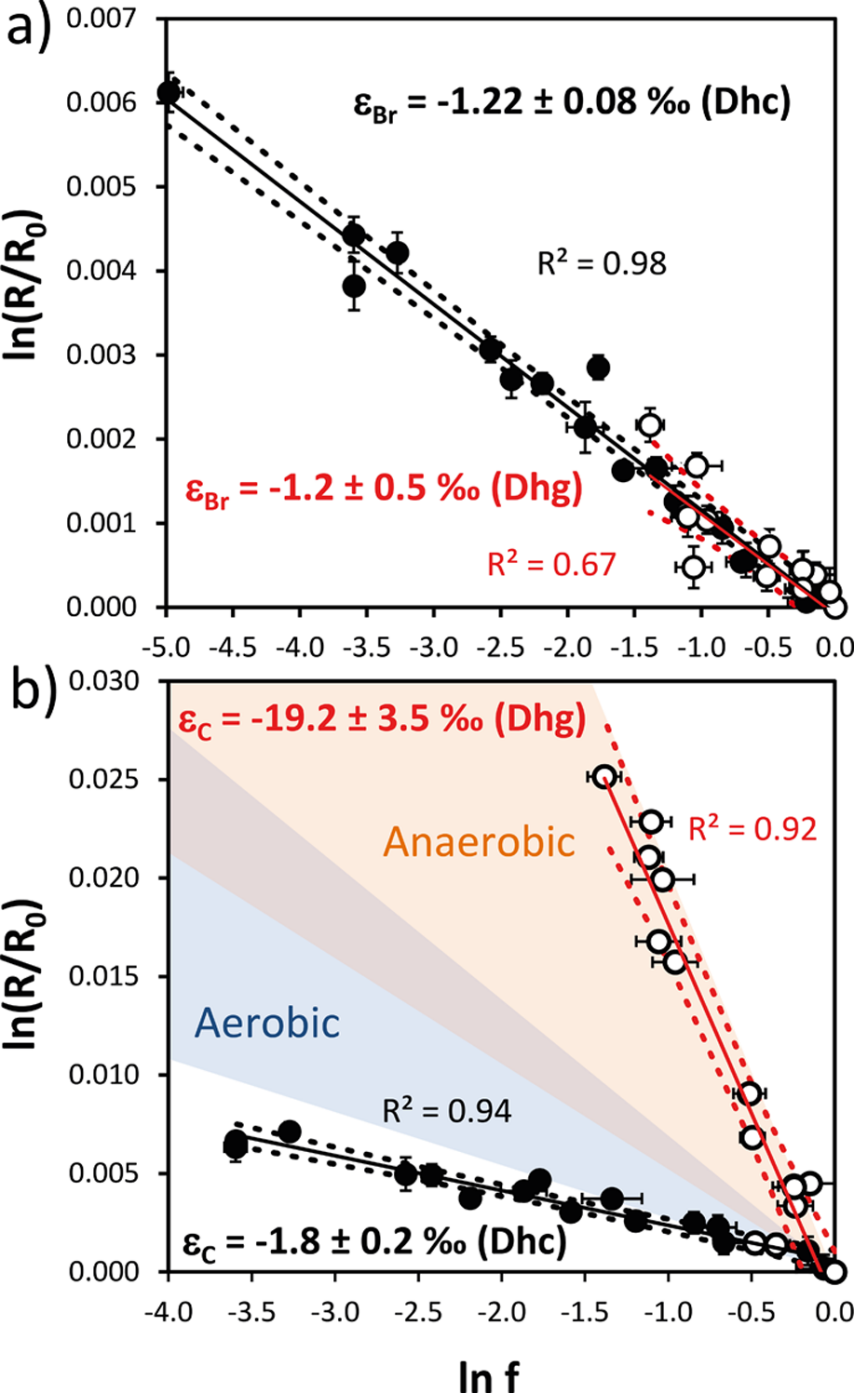
Rayleigh plots according to eq 2 of bromine (a) and carbon (b) isotopic composition of 1,2-DBA during biodegradation by *Dehalococcoides*-containing culture (Dhc, black circles) and *Dehalogenimonas*-containing culture (Dhg, white circles); *f* is the fraction of remaining 1,2-DBA. Dotted lines correspond to 95% C.I. intervals of regression parameters. The error bars for some isotope values are smaller than the symbols. Colored areas in panel (b) represent the ranges of carbon isotopic fractionation of 1,2-DBA from previous biodegradation studies conducted under aerobic (blue) and anaerobic (orange) conditions.^8,11,13^

For both microbial cultures, bromine isotope values of 1,2-DBA showed an enrichment in ^81^Br over ^79^Br during debromination following a Rayleigh isotope fractionation trend (eq 2, Figure 2a). The determined bromine isotopic fractionation (ε_bulk_^Br^) for the *Dehalococcoides*-containing microcosms (−1.22 ± 0.08‰) was very similar compared to those containing *Dehalogenimonas* (−1.2 ± 0.5‰). Low bromine isotopic effects were also observed during dihaloelimination of 1-bromo-2-chloroethane by a pure culture of *S. multivorans* (−1.7 ± 0.5‰)^35^ and hydrogenolysis of tri- and dibromoethenes by crude protein extracts of *S. multivorans* and *Desulfitobacterium hafniense* PCE-S (≤−1.3 ± 0.3‰, *n* = 6).^34^ These low bromine isotopic effects can be explained in part due to the lower relative mass difference between ^81^Br and ^79^Br (2.5%) compared, for example, to ^37^Cl and ^35^Cl (5.7% relative to ^35^Cl).^36^ This is consistent with the higher ε_bulk_^Cl^ values determined in a previous study during dichloroelimination of 1,2-dichloroethane (1,2-DCA) by similar *Dehalococcoides*-containing (−5.1 ± 0.1‰) and *Dehalogenimonas*-containing (−12.0 ± 0.8‰) enrichment cultures as used in the present study, although in that case the values were different between them.^23^

For debromination of 1,2-DBA to ethene, bromine isotope fractionation values from a previous study^11^ using reduced corrinoids (fractionation values were affected neither by transport through the cell membrane nor by interactions of the target compound with other cell materials), norpseudo-B_12_ (−3.9 ± 0.3‰) and cyano-B_12_ (−3.9 ± 0.4‰), and crude protein extract of *S. multivorans* (−2.3 ± 0.2‰) are available for comparison (Table 1). These ε_bulk_^Br^ values are higher than those measured in our study with whole cells (around −1.2‰). The weaker Br isotope fractionation observed in the experiments with intact cells could be explained by masking intrinsic isotopic effects (i.e., isotopic effects associated with the chemical bond conversion) due to microscale mass transfer limitations at microbial cell membranes.^16,24^

**Table 1:** Measured Carbon and Bromine Isotope Fractionation Values (ε_bulk_^C^ and ε_bulk_^Br^, Respectively), Dual-Element Isotope Slopes (Λ_C–Br_), and Data from Previous Studies of 1,2-DBA

experiment	conditions	ε_bulk_^C^ (‰)	*R*^2^	ε_bulk_^Br^ (‰)	*R*^2^	Λ_C–Br_	*R*^2^	references
*A. aquaticus* AD20	crude extract (oxic)	–6.9 ± 0.4	0.93	–0.6 ± 0.1	0.93	10.7 ± 2.1	0.88	Kuntze et al.^11^
*S. multivorans*	crude extract (anoxic)	–5.3 ± 0.5	0.95	–2.3 ± 0.2	0.97	2.4 ± 0.2	0.98	Kuntze et al.^11^
*Dehalococcoides*	enrichment culture (anoxic)	–1.8 ± 0.2	0.94	–1.2 ± 0.1	0.98	1.4 ± 0.2	0.95	this study
*Dehalogenimonas*	enrichment culture (anoxic)	–19.2 ± 3.5	0.92	–1.2 ± 0.5	0.67	12 ± 4	0.79	this study
norpseudo-B_12_	abiotic	–16.2 ± 1.1	0.98	–3.9 ± 0.3	0.98	4.2 ± 0.2	0.99	Kuntze et al.^11^
cyano-B_12_ type	abiotic	–16.9 ± 0.9	0.99	–3.9 ± 0.4	0.98	4.3 ± 0.4	0.96	Kuntze et al.^11^
Zn(0)	abiotic	–10.9 ± 1.1	0.97	–2.1 ± 0.3	0.96	5.3 ± 0.6	0.98	Kuntze et al.^11^
alkaline hydrolysis	abiotic (pH 8)	–29.2 ± 2.6	0.98	–1.0 ± 0.1	0.98	30.1 ± 4.2	0.98	Kuntze et al.^11^
Fenton oxidation	abiotic	–4.4 ± 0.3	0.97	not significant		∞		Kuntze et al.^11^

For carbon, isotope ratios during debromination of 1,2-DBA by the investigated microbial cultures also showed an enrichment in ^13^C over ^12^C which followed a Rayleigh trend (Figure 2b). In contrast to bromine, the ε_bulk_^C^ value for *Dehalococcoides*-containing microcosms (−1.8 ± 0.2‰) was much smaller than that for *Dehalogenimonas*-containing microcosms (−19.2 ± 3.5‰). The latter falls in the upper part of the range of ε_bulk_^C^ values reported for anaerobic biodegradation of 1,2-DBA by five different mixed cultures and crude protein extracts of *S. multivorans* (from −5.3 to −20.4‰, Figure 2b and Table S2)^8,11,13^ and is similar to those determined for abiotic dibromoelimination by corrinoid cofactors (norpseudo-B_12_: −16.2 ± 1.1‰ and cyano-B_12_: −16.9 ± 0.9‰).^11^ This suggests that the C–Br bond conversion is rate-determining and that the ε_bulk_^C^ value for the *Dehalogenimonas*-containing culture reflects the intrinsic isotope effect. Lower ε_bulk_^C^ values are generally observed for aerobic biodegradation by different bacteria (from −2.7 to −6.9‰, Figure 2b and Table S2).^11,13^ Nevertheless, the low ε_bulk_^C^ value obtained for the *Dehalococcoides*-containing culture in the present study was even lower than the range determined for aerobic biodegradation of 1,2-DBA (Figure 2b), representing the lowest ε_bulk_^C^ value determined so far for anaerobic biodegradation of 1,2-DBA. In order to investigate further the observed differences of carbon isotope fractionation during reductive dehalogenation of 1,2-DBA by enrichment cultures harboring distinct bacteria, dual-element isotope slopes and estimated AKIEs are discussed below.

### Dual C–Br Isotope Patterns

**Figure 3:**
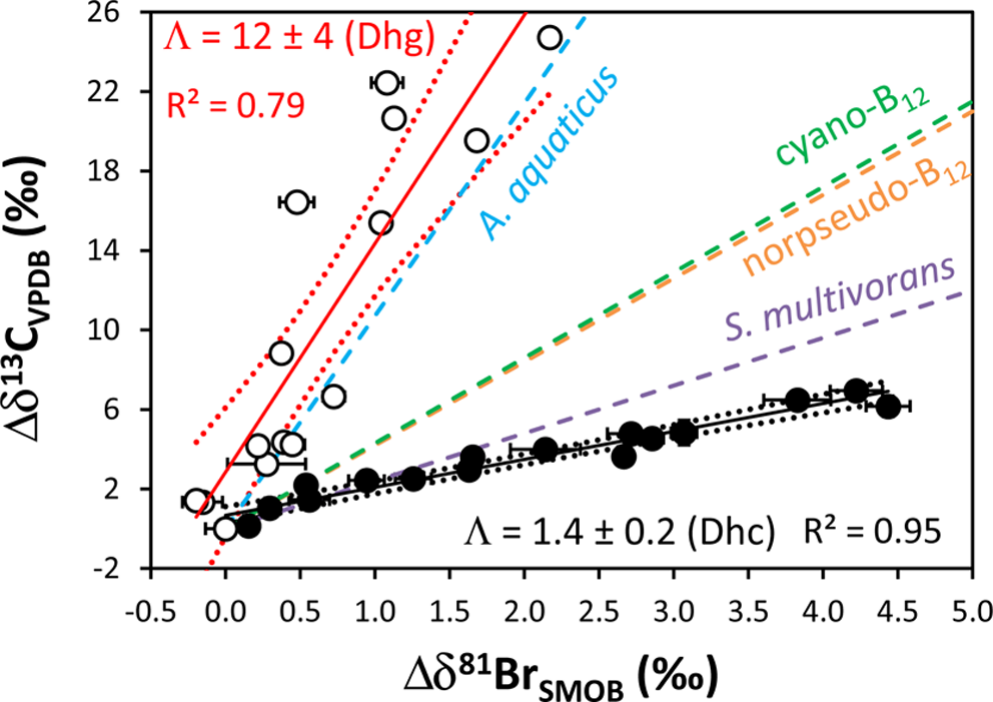
Dual C–Br isotope patterns during anaerobic biodegradation of 1,2-DBA by *Dehalococcoides*-containing culture (Dhc, black circles) and *Dehalogenimonas*-containing culture (Dhg, white circles). Dotted lines indicate the 95% C.I. of the linear regression, error bars of Δδ^13^C values are smaller than the symbols, and Λ values (±95% C.I.) are given by the slope of the linear regressions. The trend lines determined for 1,2-DBA transformation by reduced corrinoids (norpseudo-B_12_ and cyano-B_12_ types), and crude protein extracts of *S. multivorans* and *A. aquaticus* in a previous study are also indicated (dashed lines).^11^

The measured δ^81^Br and δ^13^C values of 1,2-DBA from the isotopic fractionation experiments with *Dehalococcoides*- and *Dehalogenimonas*-containing enrichment cultures were combined in a dual-element isotope plot, resulting in linear trends with strongly distinct slopes (Λ_C–Br_ = Δδ^13^C/Δδ^81^Br, stated together with 95% C.I., Figure 3 and Table 1). For *Dehalococcoides*-containing microcosms, a much smaller Λ_C–Br_ value (1.4 ± 0.2) than that of *Dehalogenimonas*-containing microcosms (12 ± 4) was observed as a result of the smaller ε_bulk_^C^ value (−1.8 ± 0.2 and −19.2 ± 3.5‰, respectively). The slope determined for *Dehalococcoides* (1.4 ± 0.2) is comparable to that obtained for experiments with crude protein extracts of *S. multivorans* (2.4 ± 0.2)^11^ in a previous study (Figure 3 and Table 1). This suggests a similar enzymatic dehalogenation mechanism and, therefore, indicates that the low ε_bulk_^C^ value for the *Dehalococcoides*-containing culture (−1.8 ± 0.2‰), compared to the crude protein extracts of *S. multivorans* (−5.3 ± 0.2‰)^11^ and the range determined from anaerobic biodegradation experiments (from −5.3 to −20.4‰, Figure 2b and Table S2),^8,11,13^ is probably due to masking of the intrinsic isotopic fractionation. This result illustrates the advantage of a dual- versus a single-element isotope approach to investigate biodegradation reactions.

The Λ_C–Br_ values reported for abiotic dibromoelimination of 1,2-DBA by reduced corrinoids (i.e., norpseudo-B_12_: 4.2 ± 0.2 and cyano-B_12_: 4.3 ± 0.4)^11^ were very different compared to that of *Dehalogenimonas* (12 ± 4) but closer to those of *Dehalococcoides* and crude protein extracts of *S. multivorans*(11) (1.4 ± 0.2 and 2.4 ± 0.2, respectively, Figure 3 and Table 1). Kuntze et al.^11^ pointed to either isotope-sensitive steps preceding the C–Br bond cleavage (e.g., compound binding to the enzyme) or differences in the reaction mechanism to explain the lower slope observed for the enzymatic reaction with crude protein extracts of *S. multivorans* compared to those obtained with pure cofactors. Similarly, previous experiments^37^ with tetrachloroethene (PCE) showed different Λ_C–Cl_ values between a crude extract of PceA of *S. multivorans* harboring different cofactors (i.e., PceA-norpseudo-B_12_: 2.2 ± 0.7 and PceA-nor-B_12_: 2.8 ± 0.5) and the respective purified corrinoids (i.e., norpseudo-B_12_: 6.9 ± 0.7 and nor-B_12_: 5.0 ± 0.8). Here, the authors suggested that the dual-element isotope slope for the dehalogenation of PCE by PceA could reflect preceding rate-limiting steps with small, but non-negligible isotope effects, during enzyme–substrate association or enzymatic structure.^37^ These results show that the characterization of dual-element isotope slopes from biodegradation experiments with intact cells and from different bacteria, as done in the present study for the first time with 1,2-DBA, is necessary for the interpretation of multi-element isotope trends in field studies. Potential reasons for the significantly different Λ_C–Br_ values during anaerobic degradation of 1,2-DBA by *Dehalococcoides*- and *Dehalogenimonas*-containing cultures are further discussed below.

### Potential Reaction Mechanisms

**Scheme 2:**
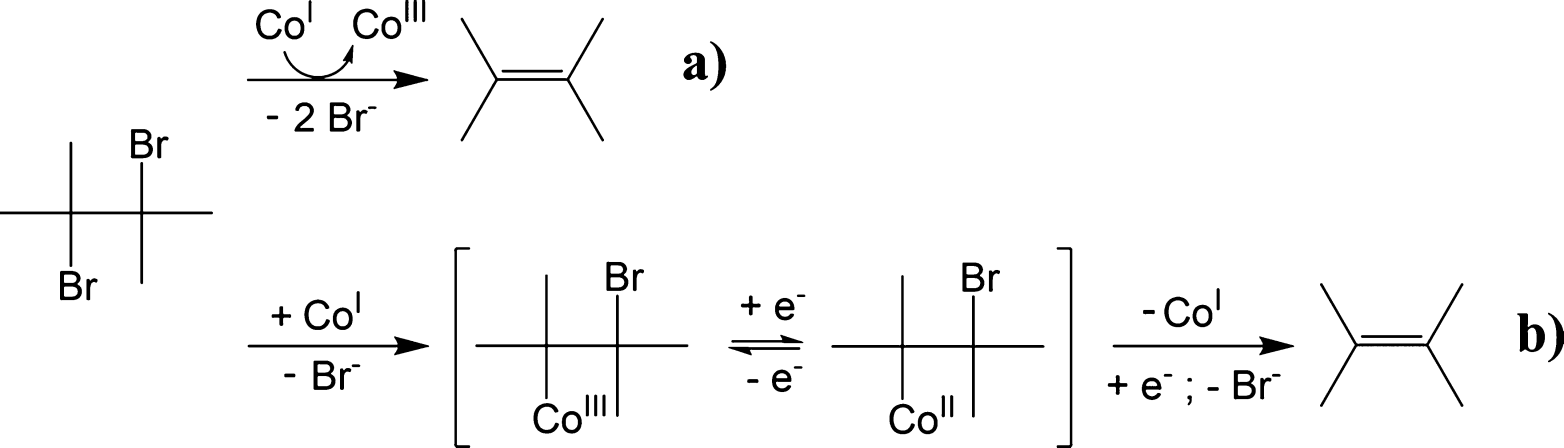
Suggested Reaction Mechanisms for 1,2-DBA Reductive Dehalogenation by *Dehalococcoides*-Containing Enrichment Culture (a) and *Dehalogenimonas*-Containing Enrichment Culture (b) in This Study: (a) Dihaloelimination and (b) Nucleophilic Substitution (S_N_2) followed by a Concerted syn-Elimination^40^

The large difference between the Λ_C–Br_ values of *Dehalogenimonas*-containing microcosms (12 ± 4) and *Dehalococcoides-*containing microcosms (1.4 ± 0.2) (Figure 3) might be interpreted as a result of different reaction pathways. Previous biodegradation studies of 1,2-DCA, the chlorinated analogue of 1,2-DBA, with *D. mccartyi* strains,^38,39^ and similar enrichment cultures of *Dehalococcoides* and *Dehalogenimonas*(23) as used in this study, suggested a 1,2-DCA transformation to ethene via concerted dihaloelimination. In addition to concerted dihaloelimination, a recent study^40^ on bacterial reduction of chlorinated alkanes, using vitamin B_12_ as a model system for RDases, suggested for the 1,2-DCA conversion to ethene an initial S_N_2-reaction followed by a concerted syn-elimination (Scheme 2b).

In the present study, insight was obtained from the comparison of Λ values for different transformation reactions. The correlation of the isotope effects in a dual-element isotope plot exhibits characteristic trends that relate to the underlying reaction mechanisms.^15,17^ In previous studies with 1,2-DCA, the Λ_C–Cl_ value reported for hydrolytic dechlorination by a pure culture of *Ancylobacter aquaticus* AD20 (7.7 ± 0.3,^17^ assumed S_N_2-reaction,^11,17,41^Scheme 1e) was very close to that determined in experiments with a *Dehalococcoides*-containing enrichment culture (6.8 ± 0.2,^23^ S_N_2-reaction^40^). The *Dehalococcoides*-containing culture was similar to the one used in the present study. The Λ_C–Cl_ value for the *Dehalococcoides*-containing culture was at that time associated with concerted dihaloelimination, but it was recently attributed to an S_N_2-reaction.^40^ Similarly, the reported Λ_C–Br_ value for hydrolytic debromination of 1,2-DBA by haloalkane hydrolytic dehalogenase (crude protein extract) of *A. aquaticus* AD20^11^ is within the uncertainty to that determined for the experiments with *Dehalogenimonas* in the present study, 11 ± 2 and 12 ± 4, respectively (Figure 3 and Table 1). Therefore, similar Λ_C–Br_ values of 1,2-DBA for debromination by *Dehalogenimonas* and hydrolytic debromination (via S_N_2) point to an underlying S_N_2-reaction mechanism during 1,2-DBA biodegradation with the *Dehalogenimonas*-containing culture. As shown in Scheme 2b, transformation of 1,2-DBA via nucleophilic substitution (S_N_2), followed by a concerted syn-elimination, result in the formation of ethene, which is consistent with the product detected in the experiments with *Dehalogenimonas* (Figure 1).

Isotopic effects from abiotic reactions are often considered close to the intrinsic isotope effects. According to studies of Zn(0)-reaction with vicinal dibromides^42^ and 1,2-DCA,^43^ a concerted (two-electron transfer) dibromoelimination of 1,2-DBA could be assumed. For 1,2-DBA, the Λ_C–Br_ value for dibromoelimination to ethene by Zn(0) from a previous study (5.3 ± 0.6)^11^ was very different compared to that of *Dehalogenimonas* (12 ± 4) but relatively similar to those obtained by the reduced corrinoids (4.2 ± 0.2 and 4.3 ± 0.4)^11^ (Table 1). As discussed above for the reduced corrinoids, the difference between the Λ_C–Br_ value of abiotic 1,2-DBA transformation by Zn(0) (5.3 ± 0.6)^11^ and those of *Dehalococcoides*-containing culture and crude extract of *S. multivorans*(11) (1.4 ± 0.2 and 2.4 ± 0.2, respectively) could be explained by potential side reactions during 1,2-DBA–enzyme association. Thus, this result would support a different reaction mechanism for 1,2-DBA debromination by the *Dehalococcoides*- and *Dehalogenimonas*-containing cultures.

Additional insight can be obtained from estimated position-specific AKIEs, provided that the chemical bond conversion in the enzyme reaction is rate-determining. Considering an S_N_2-reaction scenario for 1,2-DBA transformation by *Dehalogenimonas*, a single C–Br bond would be cleaved in the first reaction step (via S_N_2, Scheme 2b). The pronounced ε_bulk_^C^ for the *Dehalogenimonas*-containing culture (−19.2 ± 3.5‰, Figure 2b) reflects a C–Br bond cleavage as the rate-determining step. Calculated AKIEs assuming an S_N_2-reaction (AKIE^C^ = 1.040 ± 0.008 and AKIE^Br^ = 1.002 ± 0.001, see the Supporting Information) showed a good agreement with those expected from the Streitwieser semiclassical limit model for C–Br bond breakage (KIE^C^ = 1.042 and KIE^Br^ = 1.002).^11^ Therefore, the estimated AKIEs for both C and Br in the experiments with *Dehalogenimonas*-containing culture are consistent with those expected for an S_N_2-reaction.

Taken together, the different lines of evidence indicate, for the first time, the occurrence of different underlying reaction mechanisms during enzymatic 1,2-DBA transformation to ethene (i.e., S_N_2-reaction and concerted dihaloelimination, Scheme 2). This finding can explain, at least partially, the wide range of ε_bulk_^C^ values observed for anaerobic biodegradation of 1,2-DBA, from −1.8 to −20.4‰ (Figure 2b and Table S2).^8,11,13^ Our interpretation might be reinforced by the different daughter compounds detected during 1,2-DBA transformation by the distinct microbial cultures, that is ethene and VB in the experiments with *Dehalococcoides*-containing culture but only ethene with *Dehalogenimonas*-containing culture. However, further research is necessary to elucidate the source of VB. For instance, in a previous study^38^ of 1,2-DCA biodegradation by two *D. mccartyi* strains, the authors hypothesized that the traces of VC might be the result of (i) a subordinate malfunction of the involved RDase, (ii) a minor expressed additional RDase, or (iii) an abiotic reaction with reducing agents from the mineral media.

### Environmental Significance and Practical Implications for the Application of CSIA

In a recent study of 1,2-DCA biodegradation by the *D. mccartyi* strain BTF08,^39^ different Λ_C–Cl_ slopes were observed for cells with distinct RDase inventories (i.e., 5.3 ± 0.6 and 2.0 ± 0.5 for cultures with >98% TceA_btf08_ and >96% VcrA_btf08_, respectively). These dual C–Cl isotope fractionation trends correspond well with those reported for biodegradation of 1,2-DCA by similar cultures as used in the present study (i.e., 6.8 ± 0.2 and 1.89 ± 0.02 for *Dehalococcoides*- and *Dehalogenimonas*-containing cultures, respectively).^23^ In these studies,^23,39^ a concerted dihaloelimination or Co–halogen bond formation with concomitant leaving of the vicinal halogen atom was postulated as the underlying reaction mechanism. However, Heckel and Elsner,^40^ based on new data from experiments of 1,2-DCA reaction with vitamin B_12_ (Λ_C–Cl_ = 6.4 ± 0.2), hypothesized that the high Λ_C–Cl_ values of *D. mccartyi* TceA_btf08_ (5.3 ± 0.6)^39^ and *Dehalococcoides*-containing culture (6.8 ± 0.2)^23^ were associated with an S_N_2-reaction and that different reaction mechanisms were at work in these studies.

In accordance with these previous studies on 1,2-DCA, we can infer that the different reaction mechanisms determined for 1,2-DBA in the present study could probably be associated with different RDases, even if they harbor a cobalamin as a common cofactor. Importantly, the results of our study suggest that, as observed for the degradation of 1,2-DCA by *D. mccartyi* strain BTF08,^39^*Dehalogenimonas* could also be capable of employing different reaction mechanisms for the biodegradation of halogenated alkanes, that is, concerted dihaloelimination for 1,2-DCA^23,40^ and the S_N_2-reaction for 1,2-DBA. The identification of the RDases involved in the transformation of 1,2-DBA and 1,2-DCA by *Dehalogenimonas* and *Dehalococcoides* could shed more light on these questions with important insights for future biodegradation studies.^44,45^

In the last decade, single-element isotope fractionation analysis (mainly ^13^C/^12^C) has emerged as a powerful tool to evaluate the extent of halogenated organic compounds (bio)degradation in contaminated sites.^46,47^ For this approach, selection of the appropriate ε_bulk_^C^ value (or range of values), related to the ongoing degradation pathway, is crucial to get accurate estimations. Determination of ε_bulk_ values is not possible under field conditions because changes in substrate concentrations in groundwater are also related to processes other than transformation, such as hydrodynamic dispersion or sorption. The redox conditions in groundwater can be useful to constrain the ε_bulk_ values; however, this study shows that for 1,2-DBA the selection of ε_bulk_^C^ values based only on redox data could result in erroneous estimations (Figure 2b). Therefore, additional data is necessary to select adequate ε_bulk_^C^ values for quantification of 1,2-DBA biodegradation in the field.

The strongly pathway-dependent slopes Λ_C–Br_, 1.4 ± 0.2 (concerted dihaloelimination) and 12 ± 4 (S_N_2-reaction), illustrate the potential of a dual C–Br isotope analysis (i) to elucidate the reaction mechanism underlying microbial reductive dehalogenation of 1,2-DBA in the environment and (ii) to pinpoint the appropriate ε_bulk_^C^ value for the quantification of biodegradation. The similar Λ_C–Br_ values of 1,2-DBA for reductive debromination by *Dehalogenimonas* and hydrolytic debromination by *A. aquaticus* AD20 (crude protein extract)^11^ (Figure 3 and Table 1) hamper the distinction of these pathways in the field based only on C and Br isotope analysis. Hydrolytic debromination of 1,2-DBA to 2-bromoethanol (Scheme 1e) was observed under oxic conditions.^10,11^ Therefore, the combination of dual C–Br isotope data with redox conditions, product analysis (i.e., detection of ethene), and use of biomarkers may allow differentiation between hydrolytic and reductive debromination of 1,2-DBA in the environment. Engineered abiotic transformation reactions of 1,2-DBA such as Fenton oxidation and alkaline hydrolysis (pH 8) showed much higher Λ_C–Br_ values (i.e., ∞ and 30.1 ± 4.2, respectively, Table 1)^11^ compared to the biodegradation reactions investigated in this study.

The results of this study provide valuable information for future application of CSIA to investigate 1,2-DBA biodegradation in contaminated sites, contributing to the urgent need of reducing uncertainties in the quantification of compound transformation. In addition, the results suggest that the combination of dual C–Br isotope analysis with molecular biology tools might be used to characterize anaerobic biodegradation of 1,2-DBA by different bacteria.

## Supporting Information Available

Further information about materials and methods, including DNA extraction and 16S rRNA gene sequencing/qPCR, activity assays, batch experiments, C and Br isotope analysis, and calculation of AKIEs; product concentration pattern during biodegradation of 1,2-DBA by *Dehalococcoides*; microbial composition of the *Dehalogenimonas* enrichment culture; plot of ethene production and bromide release against 1,2-DBA consumed; and comparison of literature and experimental carbon isotope fractionation values of 1,2-DBA (PDF)
