# Supplementary material for: Dual C–Br Isotope Fractionation Indicates Distinct Reductive Dehalogenation Mechanisms of 1,2-Dibromoethane in Dehalococcoides- and Dehalogenimonas-Containing Cultures
Source: Environ Sci Technol. 2023 Jan 26;57(5):1949–58. doi: 10.1021/acs.est.2c07137 (PMC9910042; doi:10.1021/acs.est.2c07137)
Supplement: Supplementary file 1 — es2c07137_si_001.pdf [file es2c07137_si_001.pdf]

Supporting Information

Dual C-Br isotope fractionation indicates distinct reductive dehalogenation mechanisms of 1,2-Dibromoethane in *Dehalococcoides*- and *Dehalogenimonas*-containing cultures

Jordi Palau,[†*] *Alba Trueba-Santiso,*[§,1] *Rong Yu,*[γ] *Siti Hatijah Mortan,*[§,2], *Orfan Shouakar-Stash,*[╪] *David L. Freedman,*[‖] *Kenneth Wasmund,*[╬] *Daniel Hunkeler,*[┤] *Ernest Marco-Urrea,*[§*] *Monica Rosell*[†]

† Grup MAiMA, SGR Mineralogia Aplicada, Geoquímica i Geomicrobiologia, Departament de Mineralogia, Petrologia i Geologia Aplicada, Facultat de Ciències de la Terra, Institut de Recerca de l'Aigua (IdRA), Universitat de Barcelona (UB), Martí Franquès s/n, Barcelona 08028, Spain.
§ Departament d'Enginyeria Química, Biològica i Ambiental, Universitat Autònoma de Barcelona (UAB), Carrer de les Sitges s/n, Bellaterra 08193, Spain.
γ Synterra Corporation, Greenville, South Carolina 29601, United States.
╪ Isotope Tracer Technologies Inc., 608 Weber St. N., Unit #3, Waterloo, Ontario, N2V 1K4, Canada.
‖ Department of Environmental Engineering and Earth Sciences, Clemson University, Clemson, South Carolina 29634, United Sates.
╬ Division of Microbial Ecology, Centre for Microbiology and Environmental Systems Science, University of Vienna, Vienna, A-1030, Austria.
┤ Centre for Hydrogeology and Geothermics, University of Neuchâtel, Neuchâtel 2000, Switzerland.

[1] Present address: CRETUS Institute, Department of Chemical Engineering, Universidade de Santiago de Compostela, 15782 Santiago de Compostela, Galicia, Spain
[2] Present address: Faculty of Chemical and Process Engineering Technology, Universiti Malaysia Pahang, Lebuhraya Tun Razak, Gambang, 26300 Kuantan, Pahang, Malaysia.

Content (14 Pages)

Supplementary Materials and Methods S3

- SMM1. DNA extraction and 16S rRNA gene amplicon sequencing
- SMM2. DNA extraction and real-time PCR (qPCR)
- SMM3. Activity assays with cell suspensions of *Dehalogenimonas*
- SMM4. Batch experiments with the *Dehalococcoides*-containing culture
- SMM5. Batch experiments with the *Dehalogenimonas*-containing culture
- SMM6. ^{13}C- and ^{81}Br-CSIA of 1,2-DBA
- SMM7. Calculation of apparent kinetic isotope effects (AKIEs)

Supplementary Results S9

- SR1. Product concentration pattern during transformation of 1,2-DBA by *Dehalococcoides*.

Supplementary Figures S10

Supplementary Tables S12

References S13

Supplementary Materials and Methods

SMM1. DNA extraction and 16S rRNA gene amplicon sequencing

Cells were harvested by centrifugation (7000 g, 40 min at 10 ºC) from 65-mL samples (one microcosm) using sterile falcon tubes in an Avanti J-20 centrifuge. The pelleted cells were resuspended in sterile phosphate-buffered saline (PBS) buffer and DNA was then extracted using the Gentra Puregene Yeast/Bact kit (Qiagen) following the instructions from the manufacturer. The DNA of each of the biological replicates was extracted and analyzed separately. Amplicons of the region V3-V4 for 16S rRNA genes were amplified with primers S-D-Bact-0341-b-S-17/S-D-Bact-0785-a-A-21[1] using the Illumina MiSeq platform at Serveis de Genòmica i Bioinformàtica from the Universitat Autònoma de Barcelona (UAB). Gene sequences were processed using the 16S Metagenomics workflow in the MiSeq Reporter analysis software based on quality scores generated by real-time analysis during the sequencing run. Quality filtered indexed reads were demultiplexed for generation of individual FASTQ files and aligned using the banded Smith-Waterman method[2] of the Illumina-curated version of the Greengenes taxonomic database. The output of this workflow was a classification of reads at multiple taxonomic levels. To calculate relative abundance, we divided the number of sequences belonging to a taxonomic level by the total number of sequences obtained from the specific sample.

SMM2. DNA extraction and real-time PCR (qPCR)

Cells were harvested by centrifugation (7000 g, 10 min at 4 ºC) from triplicate 1-mL samples collected from duplicate *Dehalogenimonas*-containing microcosms at time zero and after the consumption of 100 µM 1,2-DBA. The DNA was then extracted from the samples using the Gentra Puregene Yeast/Bact kit (Qiagen) and following the instructions from the manufacturer.

Real-time PCR reactions targeting 16S rRNA genes of the genus *Dehalogenimonas* (20 µL total) included 10 µL iQ™ SYBR® Green Supermix (BioRad), 0.5 µM of each primer, 8.4 µL ddH20 and 1 µL of DNA. PCR cycling was: 95 °C for 3 min, followed by 40 cycles of 95 °C for 30 s, 63 °C for 30 s, and 72 °C for 30 s, and a final extention at 72 °C for 5 min. The primers

used were DHG-2-F (5'-GGYACAATGGGTTGCCACCGG-3') and DHG-2-R (5'-AACGCGCTATGCTGACACGCGT-3').[3] DNA standards included a 16S rRNA gene cloned from *Dehalogenimonas alkenigignens* strain BRE15M that was cloned into a pPCR4-TOPO vector (Invitrogen, USA). Clone inserts were amplified using PCR with M13 primers, then PCR purified and diluted from 10^8 to 10^2 copies per µL. Real-time PCR assays were done in triplicate with a CFX96 Touch™ Real-Time PCR Detection System (version 3.1) (Bio-Rad). Melt curve analysis was done using default instrument settings, increasing from 65 to 95 °C.

SMM3. Activity assays with cell suspensions of *Dehalogenimonas*

To test reductive dehalogenase enzyme activity, specific assays were set-up inside an anoxic glovebox. Anoxic 10 mL glass vials were used, containing 2 mL of an assay buffer with 200 mM potassium acetate buffer (pH 5.8), 2 mM methyl viologen, and 2 mM titanium (III) citrate [2 mM in respect to titanium (III)], the corresponding brominated compound (i.e., 1,2-DBA or VB), and 200 µL of a 3×10^7 cells mL^{-1} concentrate, quantified by direct epifluorescence microscopy.

SMM4. Batch experiments with the *Dehalococcoides*-containing culture

The chemicals (purity, sources) used were 1,2-DBA (99%, Acros Organics), polymer grade ethene (99.9%, Airgas), ethane (99.95%, Matheson), methane (99%, Matheson), bromoethane (99%, EMD Chemicals), and VB (98%, Pfaltz & Bauer). Sodium lactate syrup was obtained from EM Science (58.8 to 61.2% sodium lactate; specific gravity = 1.31). All other chemicals were reagent grade. Enrichment cultures were grown in a mineral salts medium, as described in Yu et al.[4]

The *Dehalococcoides* enrichment culture was developed with 1,2-DBA as the terminal electron acceptor and lactate as the electron donor. The inoculum was an enrichment culture that uses all of the chlorinated ethenes as terminal electron acceptors and lactate as the electron donor, as described in Yu et al.[4] Sodium hydroxide was added periodically to keep the pH between 6.7 and 7.1. Prior to its use in the current study, the 1,2-DBA enrichment culture was maintained for more than nine years on 1,2-DBA and lactate. The rate at which 1,2-DBA was consumed remained similar to the behavior shown in the Supporting Information from Yu et al.[4], i.e., ~19 µM/d. The 1,2-DBA enrichment

stoichiometrically reduces 1,2-DBA to ethene as the primary product, along with trace levels of VB.

The Supporting Information for Palau et al.[5] describes characterization of the chloroethene respiring culture that was used to create enrichment cultures that use 1,2-dichloroethane (DCA) and 1,2-DBA as terminal electron acceptors. Briefly, Illumina 16S rRNA gene sequencing was performed and indicated that the presence of *Dehalococcoides* (5.0%) was much larger compared to *Desulfitobacterium* (0.051%) and *Dehalobacter* (0.39%). *Dehalogenimonas* was not detected. For the 1,2-DBA enrichment culture, Yu et al.[4] reported that *Dehalococcoides* increased in copy numbers during reduction of 1,2-DBA to ethene, while *Dehalobacter* and *Desulfitobacterium* did not.

For the 1,2-DBA isotope experiment, a total of 27 serum bottles (plus 3 controls) were prepared by dispensing 65 mL of the enrichment culture. 1,2-DBA was added as a water-saturated solution (800 µL per bottle), resulting in an initial aqueous phase concentration of ~50 mg/L (when taking into account partitioning between the headspace and liquid using Henry's law). Sodium lactate was added to ensure an excess of electron equivalents for dehalogenation (70 µL of a Na-lactate stock solution containing 456.2 g/L of 60% Na-lactate syrup).[4] The *Dehalococcoides*-containing microcosms were incubated at room temperature (22-24 °C) in an inverted position on a shaker table, to keep the liquid phase completely mixed at all times.

The amounts of 1,2-DBA, VB and ethene in the *Dehalococcoides*-containing microcosms were measured at Clemson University (CU) laboratories using a gas chromatograph-flame ionization detector (GC-FID) (Hewlett Packard 5890 Series II). Further information is available in a previous study.[4] Briefly, aqueous phase detection limits were 0.021, 0.001 and 0.0004 µmol/bottle (60, 1.1 and 0.024 µg/L), respectively. The GC-FID response to a headspace sample (0.5 mL) was calibrated to give the total mass of the compound in that bottle,[6] which was then converted to an aqueous-phase concentration using Henry's law constants.[7] The stoichiometry of 1,2-DBA biodegradation accounted for the amounts that carried over from the enrichment culture, i.e., which are approximately 1.0 ± 0.2 µmol/bottle (62 ± 10 µg/L) ethene and 0.005 ± 0.002 µmol/bottle (6.3 ± 1.9 µg/L) VB.

SMM5. Batch experiments with the *Dehalogenimonas*-containing culture

A *Dehalogenimonas*-containing culture transforming 1,2-DCP (50 μM) to propene was maintained for more than five years in the UAB laboratory as described elsewhere.[8] A total of 18 serum bottles (including 2 controls) containing the enriched culture of *Dehalogenimonas* were prepared in a defined medium described elsewhere.[8] Briefly, each microcosm contained 65 mL of anaerobic bicarbonate-buffered medium, reduced with $Na_2S \times 9H_2O$ and L-cysteine (0.2 mM each) with 5 mM sodium acetate as carbon source, and gassed with N_2/CO_2 (4:1, v/v, 0.2 bar overpressure) and H_2 (added to an overpressure of 0.4 bar). All microcosms were incubated under static conditions in the dark at 25 ºC in a thermostatic chamber.[9]

The halogenated compounds and ethene concentrations were analyzed from headspace samples with a GC-FID (Agilent 6890N) equipped with an HP-5 column (30 m × 0.32 mm with 0.25 μm film thickness; Agilent Technologies). Helium was used as the carrier gas (1.5 mL min^{-1}). The injector and detector temperatures were both set at 250 °C. After the injection of the sample (split ratio = 2:1), the initial oven temperature (40 °C), ramped at 10 °C min^{-1} to 50 °C, and then ramped at 20 °C min^{-1} to 120 °C. Calibration of halogenated compounds and ethene was based on aqueous standards, with the same liquid and headspace volumes as in the microcosms. The solutions were allowed to equilibrate overnight, and afterwards they were analyzed in the GC-FID as described above. Results are presented as nominal concentrations (μmol per L of liquid volume).

SMM6. ^{13}C- and ^{81}Br-CSIA of 1,2-DBA

Samples from the microcosms were analyzed for carbon isotope ratios at the Scientific and Technological Centers of the University of Barcelona (CCiT-UB) using a GC (Agilent 6890, Palo Alto, CA, USA) coupled to an IRMS (Delta Plus, Thermo Finnigan, Bremen, Germany). Liquid aliquots were diluted to a similar 1,2-DBA concentration in 20 mL vials containing a 30 mm PTFE-coated stir bar. The vials were immediately sealed with PTFE/Silicone septa and aluminum crimp caps. Then, the sample solution was stirred at room temperature and 1,2-DBA was extracted during 20 min by headspace solid phase micro extraction (SPME) using a manual sampler holder equipped with a 75 μm Carboxen-PDMS fiber (Supelco, Bellefonte, PA). The GC was equipped with a Supelco SPB-624 column (60 m × 0.32 mm, 1.8 μm film thickness; Bellefonte, PA). The injector was set at 220 °C in split mode (1:10) and the oven temperature program was kept at 60

°C for 2 min, heated to 220 °C at a rate of 8 °C min^{-1} and finally held at 220 °C for 5 min. Helium was used as a carrier gas with a gas flow rate of 1.8 mL min^{-1}.

An isotopic working standard of 1,2-DBA (≥ 98.0 %, Sigma-Aldrich) was used to ensure accuracy of the isotopic measurements during the course of samples analysis. The isotopic signature of the working standard ($\delta^{13}C_{VPDB}$ = -28.93 ± 0.05‰, ±1σ, n = 10) was determined beforehand by an elemental analyzer coupled to an IRMS. Several 1,2-DBA aqueous isotopic standards were prepared daily from the same pure 1,2-DBA (stock solutions were prepared first in HPLC grade methanol) and analyzed on the same days as the samples. Standards and samples were analyzed by duplicate as a quality control.

Samples for bromine isotope analysis were measured at Isotope Tracer Technologies Inc. (Canada) using a GC (Agilent 6890, Santa Clara, CA, USA) coupled to an IRMS (MAT 253, Thermo Finnigan, Bremen, Germany). This IRMS was equipped with nine collectors and the bromine isotope analyses were performed using a continuous flow IRMS configuration with a dual-inlet (DI) mode option. The DI bellows are used as the monitoring gas reservoir and reference peaks were introduced at the beginning of each analysis run.

Samples and isotopic standards were prepared in 20 mL vials and sealed with crimped septa caps (PTFE/Silicone). These vials contain 16 mL of solution and 4 mL of headspace. The 1,2-DBA in solution was extracted by headspace SPME (75 μm Carboxen-PDMS for Merlin Microseal™, 23 gauge needle holder from Supelco, Bellefonte, PA, US) using a CombiPAL SPME autosampler (CTC Analytics, Zwingen, Switzerland). The SPME fiber was desorbed into the GC inlet at 270 °C. The GC was equipped with a DB-5 MS column (60 m × 0.32 mm × 1 μm; Agilent). The carrier gas (He) flow rate of the GC was set at 1.8 mL min^{-1} and the oven temperature was programmed as follows: 40 °C (9 min), ramp at 10 °C min^{-1} to 100 °C and ramp at 46 °C min^{-1} to 250 °C (6.75 min).

Two pure 1,2-DBA isotopic working standards were used for instrument monitoring and external calibration of sample raw $\delta^{81}Br$ values to the international standard mean ocean bromide (SMOB) scale. The analysis scheme for the samples calibration with respect to SMOB scale consists of a sequence of samples bracketed by two sets of standards, one set at the beginning of the run and another set at the end of the analysis. Each set of standards comprise six replicates for each of the two standards in varying concentrations, and each run is composed of 12 to 20 samples. Blanks were included at the beginning of each run and carry-over was not observed.

SMM7. Calculation of apparent kinetic isotope effects (AKIEs)

Intrinsic KIEs are position specific whereas ε_{bulk} values are calculated from compound-average isotope data (eq. 2 in the main text). Therefore, observable ε_{bulk} values have to be converted into AKIEs in order to obtain information about the underlying reaction mechanisms.[10] For the calculation and interpretation of AKIEs a hypothesis about the reaction mechanism, or assumed reaction mechanism, is necessary. The effects of non-reacting positions within the molecule, as well as of intramolecular competition, are then taken into account using eq. S1 and S2, respectively,[10]

$$\varepsilon_{rp} \approx \frac{n}{x} \cdot \varepsilon_{bulk} \tag{S1}$$

$$\text{AKIE} = \frac{1}{z \cdot \varepsilon_{rp} + 1} \tag{S2}$$

where ε_{rp} is the isotopic fractionation at the reactive position, "n" is the number of atoms of the element considered, "x" is the number of reactive sites and "z" the number of identical reactive sites undergoing intramolecular competition. These equations assume the absence of secondary isotope effects. For carbon, secondary isotope effects are usually insignificant.[10] In symmetric molecules such as 1,2-DBA, all atoms are in equivalent reactive positions (n = x), and therefore, ε_{rp} is directly obtained from the slopes of the Rayleigh plots (Fig. 2). If the two C-Br bonds are broken in sequence (e.g., S_N2-reaction), assuming that the first bond cleavage is the rate-determining step, then z = 2 in eq. S2 as both C-Br bonds compete for reaction.

Supplementary Results

SR1. Product concentration pattern during transformation of 1,2-DBA by *Dehalococcoides.*

In order to investigate the biodegradation products of 1,2-DBA in the experiments with *Dehalococcoides*, data collected by GC-FID measurement on single bottles that continued to be incubated (headspace monitoring) are shown in **Figure S3**. Five microcosms were selected based on their similar 1,2-DBA degradation rate and extent, but similar concentration patterns were observed for all the microcosms.

The expected rate of 1,2-DBA degradation by abiotic hydrolysis and dehydrohalogenation at the incubation temperature of the experiments with *Dehalococcoides* (23 ºC) can be estimated using the Arrhenius equation, eq. S3,

$$k = A \cdot \exp(-Ea/RT) \qquad \text{(S3)}$$

where "k" is the first order rate constant (h^{-1}), "A" is the frequency factor (h^{-1}), "R" is the gas constant (8.314×10^{-3} kJ mol^{-1} K^{-1}), "Ea" is the activation energy (kJ mol^{-1}) and "T" is the absolute temperature (K). According to the "Ea" (112 kJ mol^{-1}) and "A" (8.7×10^{14} h^{-1}) values determined by Groos et al.[11] the transformation rate is estimated to be 1.53×10^{-5} h^{-1} (i.e., half-live of around 5 years). This rate was used to simulate the remaining amount of 1,2-DBA and the formation of VB considering hydrolysis/dehydrohalogenation as the sole reaction mechanism (**Fig. S3**). Groos et al.[11] (and references herein) indicated that 1,2-DBA dehydrohalogenation to VB accounted for less than 5% of 1,2-DBA degradation. Accordingly, in the present study the formation of VB was simulated assuming that it represents a 5% of the total 1,2-DBA transformed. Using the parameters indicated above, a very low degradation rate of 1,2-DBA by hydrolysis/dehydrohalogenation is obtained at 4 ºC (half-live of approximately 116 years) indicating that insignificant transformation of 1,2-DBA occurred during the samples storage.

Supplementary Figures

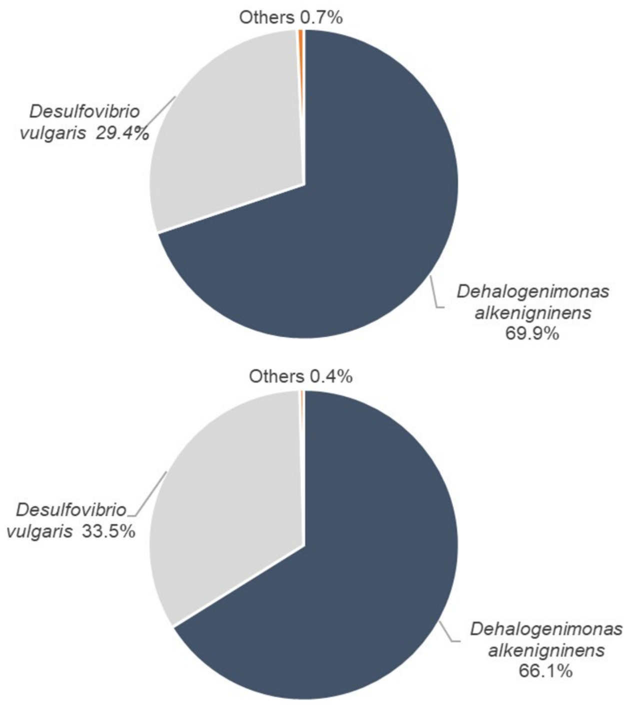

Figure S1. Microbial composition of the *Dehalogenimonas*-containing culture after Illumina sequencing of the 16S rRNA gene. Each pie chart illustrates an independent enrichment culture.

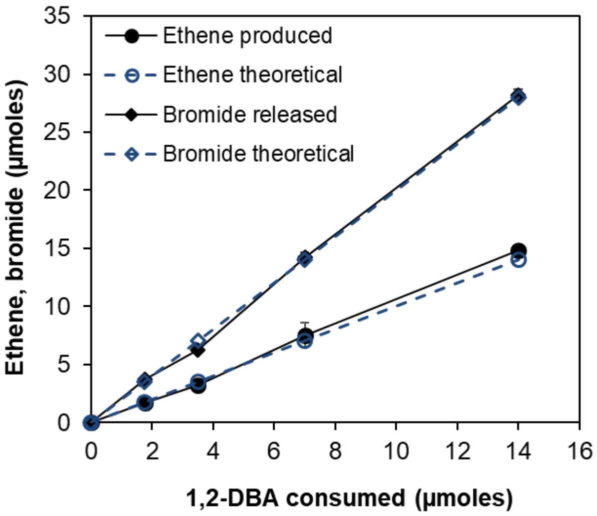

Figure S2. Ethene production and bromide release against the micromoles of 1,2-DBA consumed over the time in the microcosms depicted in Figure 1. The theoretical concentration of ethene and bromide was calculated based on the stoichiometry of the 1,2-DBA consumed. Error bars represent the standard deviation for the values of biological triplicates.

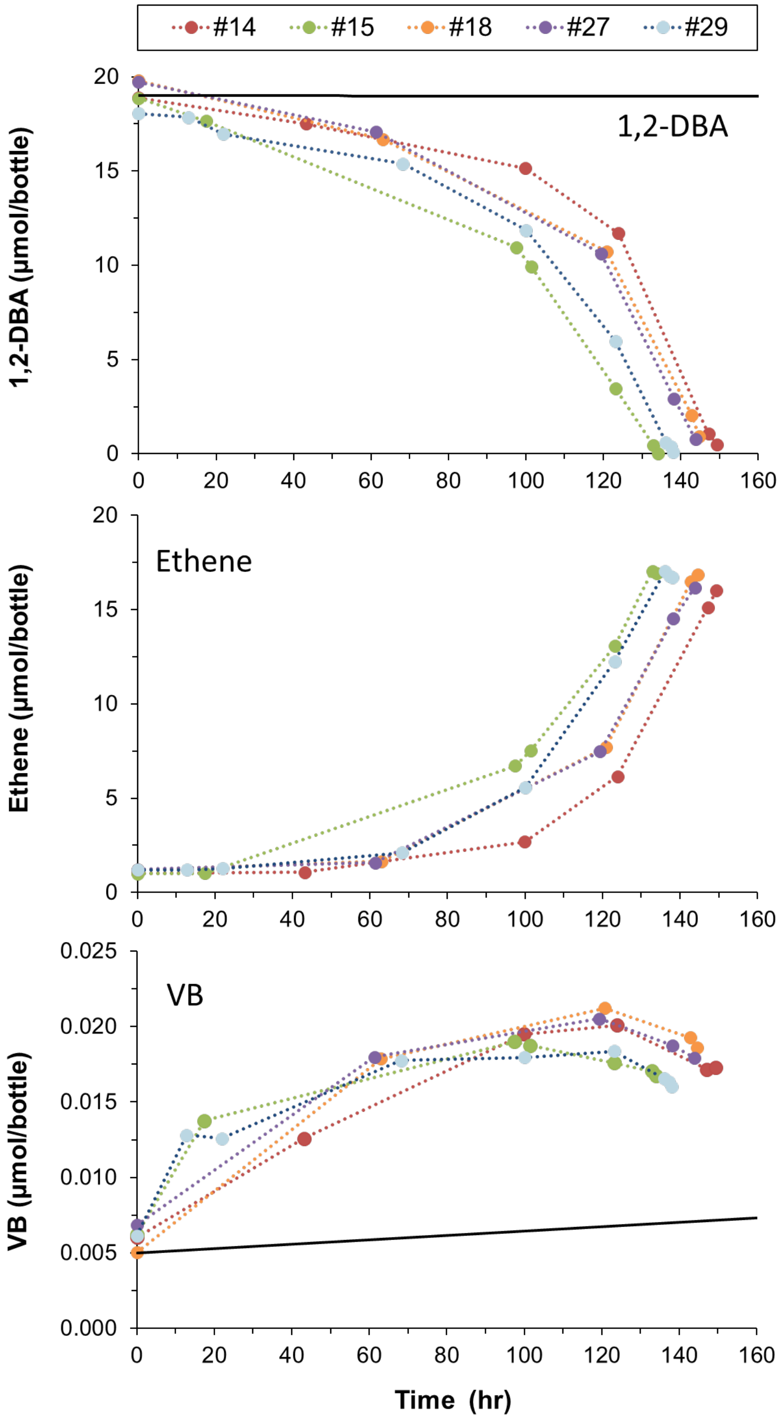

Figure S3. Biodegradation of 1,2-DBA and accumulation of daughter products in batch experiments with *Dehalococcoides*-containing microcosms. Five of 30 bottles are shown. Data points show the total amounts per bottle and directly reveal the stoichiometry of 1,2-DBA conversion to ethene and VB. Solid lines indicate the amounts expected for abiotic transformation of 1,2-DBA via parallel hydrolysis and dehydrohalogenation (upper panel) and formation of VB via dehydrohalogenation (lower panel), assuming abiotic hydrolysis/dehydrohalogenation as the sole reaction mechanism.

Supplementary Tables

Table S1. Activity test with cell suspensions from the *Dehalogenimonas*-containing culture. Abiotic control (AC): controls containing medium and 1,2-DBA without cells; biotic control (BC): controls containing medium and cells without 1,2-DBA; experimental vials (EV). Ethene in BC is derived from the inoculum. The vials were incubated for 24 hours and except BC were amended with 50 μM 1,2-DBA. Values are averages of duplicate samples.

	**1,2-DBA (μM)**	**Ethene (μM)**	**Sum of 1,2-DBA and ethene (μM)**
AC 1	47.2	0	47.2
AC 2	49.8	0	49.8
BC 1	0	1.9	1.9
BC 2	0	1.6	1.6
EV 1	33.8	20.9	54.7
EV 2	34.8	15.7	50.5

Table S2. Literature carbon isotope fractionation values (ε^{C}_{bulk}) of 1,2-DBA from laboratory biodegradation studies, under anoxic and oxic conditions, and results from this study.

**Experiment**	**ε^{C}_{bulk} (‰)**	**R^2**	**Conditions**	**Reference**
SDC-9 (Mixed culture)[a]	-8.8 ± 0.4	0.99	Anoxic	Groos et al.[11]
Hawaii-05 (Mixed culture)[a]	-7.6 ± 0.5	0.99	Anoxic	Groos et al.[11]
PJKS (Mixed culture)[a]	-20.4 ± 2.9	0.95	Anoxic	Groos et al.[11]
ENV490 (Mixed culture)[b]	-8.8 ± 1.1	0.96	Anoxic	Groos et al.[11]
Mixed culture[b]	-5.6 ± 1	0.99	Anoxic	Henderson et al.[12]
Sulfurospirillum multivorans (Crude extract)	-5.3 ± 0.5	0.95	Anoxic	Kuntze et al.[13]
Dehalococcoides (Enrichment culture)	-1.8 ± 0.2	0.94	Anoxic	This study
Dehalogenimonas (Enrichment culture)	-19.2 ± 3.5	0.92	Anoxic	This study
Ancylobacter aquaticus AD20 (Crude extract)	-6.9 ± 0.4	0.93	Oxic	Kuntze et al.[13]
Methylocella palustris	-2.7 ± 0.2	0.99	Oxic	Groos et al.[11]
Mycobacterium sphagni ENV482	-5.8 ± 1.0	0.91	Oxic	Groos et al.[11]

[a] Cultures produced for large scale bioremediation treatment. [b] Field derived microcosms.

REFERENCES

(1) Klindworth, A.; Pruesse, E.; Schweer, T.; Peplies, J.; Quast, C.; Horn, M.; Glöckner, F. O. Evaluation of General 16S Ribosomal RNA Gene PCR Primers for Classical and Next-Generation Sequencing-Based Diversity Studies. *Nucleic Acids Res* **2013**, *41* (1). https://doi.org/10.1093/NAR/GKS808.

(2) Smith, T. F.; Waterman, M. S. Identification of Common Molecular Subsequences. *J Mol Biol* **1981**, *147* (1), 195–197. https://doi.org/10.1016/0022-2836(81)90087-5.

(3) Chen, J.; Bowman, K. S.; Rainey, F. A.; Moe, W. M. Reassessment of PCR Primers Targeting 16S RRNA Genes of the Organohalide-Respiring Genus *Dehalogenimonas*. *Biodegradation* **2014**, *25* (5), 747–756. https://doi.org/10.1007/S10532-014-9696-Z.

(4) Yu, R.; Peethambaram, H. S.; Falta, R. W.; Verce, M. F.; Henderson, J. K.; Bagwell, C. E.; Brigmon, R. L.; Freedman, D. L. Kinetics of 1,2-Dichloroethane and 1,2-Dibromoethane Biodegradation in Anaerobic Enrichment Cultures. *Appl Environ Microbiol* **2013**, *79* (4), 1359–1367. https://doi.org/Doi 10.1128/Aem.02163-12.

(5) Palau, J.; Yu, R.; Hatijah Mortan, S.; Shouakar-Stash, O.; Rosell, M.; Freedman, D. L.; Sbarbati, C.; Fiorenza, S.; Aravena, R.; Marco-Urrea, E.; Elsner, M.; Soler, A.; Hunkeler, D. Distinct Dual C-Cl Isotope Fractionation Patterns during Anaerobic Biodegradation of 1,2-Dichloroethane: Potential to Characterize Microbial Degradation in the Field. *Environ Sci Technol* **2017**, *51* (5), 2685–2694. https://doi.org/10.1021/acs.est.6b04998.

(6) Verce, M. F.; Ulrich, R. L.; Freedman, D. L. Characterization of an Isolate that Uses Vinyl Chloride as a Growth Substrate under Aerobic Conditions. *Appl Environ Microbiol* **2000**, *66* (8), 3535–3542.

(7) Gossett, J. M. Measurement of Henry's Law Constants for C1 and C2 Chlorinated Hydrocarbons. *Environ Sci Technol* **1987**, *21* (2), 202–208. https://doi.org/10.1021/es00156a012.

(8) Martin-Gonzalez, L.; Hatijah Mortan, S.; Rosell, M.; Parlade, E.; Martinez-Alonso, M.; Gaju, N.; Caminal, G.; Adrian, L.; Marco-Urrea, E. Stable Carbon Isotope Fractionation During 1,2-Dichloropropane-to-Propene Transformation by an Enrichment Culture Containing *Dehalogenimonas* Strains and a DcpA Gene. *Environ Sci Technol* **2015**, *49* (14), 8666–8674. https://doi.org/10.1021/acs.est.5b00929.

(9) Löffler, F. E.; Sanford, R. A.; Ritalahti, K. M. Enrichment, Cultivation, and Detection of Reductively Dechlorinating Bacteria. *Methods Enzymol* **2005**, *397*, 77–111. https://doi.org/10.1016/S0076-6879(05)97005-5.

(10) Elsner, M.; Zwank, L.; Hunkeler, D.; Schwarzenbach, R. P. A New Concept Linking Observable Stable Isotope Fractionation to Transformation Pathways of Organic Pollutants. *Environ Sci Technol* **2005**, *39* (18), 6896–6916. https://doi.org/Doi 10.1021/Es0504587.

(11) Groos, P. G. K. van; Hatzinger, P. B.; Streger, S. H.; Vainberg, S.; Philp, R. P.; Kuder, T. Carbon Isotope Fractionation of 1,2-Dibromoethane by Biological and Abiotic Processes. *Environ Sci Technol* **2018**, *52* (6), 3440–3448. https://doi.org/10.1021/ACS.EST.7B05224.

(12) Henderson, J. K.; Freedman, D. L.; Falta, R. W.; Kuder, T.; Wilson, J. T. Anaerobic Biodegradation of Ethylene Dibromide and 1,2-Dichloroethane in the Presence of Fuel Hydrocarbons. *Environ Sci Technol* **2008**, *42* (3), 864–870. https://doi.org/10.1021/ES0712773.

(13) Kuntze, K.; Kozell, A.; Richnow, H. H.; Halicz, L.; Nijenhuis, I.; Gelman, F. Dual Carbon–Bromine Stable Isotope Analysis Allows Distinguishing Transformation Pathways of Ethylene Dibromide. *Environ Sci Technol* **2016**, *50* (18), 9855–9863. https://doi.org/10.1021/ACS.EST.6B01692.
